# *In vitro* Analysis of the Intradiscal Pressure of the Thoracic Spine

**DOI:** 10.3389/fbioe.2020.00614

**Published:** 2020-06-17

**Authors:** Hans-Joachim Wilke, Andrea Herkommer, Karin Werner, Christian Liebsch

**Affiliations:** Institute of Orthopaedic Research and Biomechanics, Trauma Research Centre Ulm, Ulm University, Ulm, Germany

**Keywords:** thoracic spine, intradiscal pressure, human intervertebral disc, *in vitro* study, biomechanics

## Abstract

The hydrostatic pressure of the nucleus pulposus represents an important parameter in the characterization of spinal biomechanics, affecting the segmental stability as well as the stress distribution across the anulus fibrosus and the endplates. For the development of experimental setups and the validation of numerical models of the spine, intradiscal pressure (IDP) values under defined boundary conditions are therefore essential. Due to the lack of data regarding the thoracic spine, the purpose of this *in vitro* study was to quantify the IDP of human thoracic spinal motion segments under pure moment loading. Thirty fresh-frozen functional spinal units from 19 donors, aged between 43 and 75 years, including all segmental levels from T1–T2 to T11–T12, were loaded up to 7.5 Nm in flexion/extension, lateral bending, and axial rotation. During loading, the IDP was measured using a flexible sensor tube, which was inserted into the nucleus pulposus under x-ray control. Pressure values were evaluated from third full loading cycles at 0.0, 2.5, 5.0, and 7.5 Nm in each motion direction. Highest IDP increase was found in flexion, being significantly (*p* < 0.05) increased compared to extension IDP. Median pressure values were lowest in lateral bending while exhibiting a large variation range. Flexion IDP was significantly increased in the upper compared to the mid- and lower thoracic spine, whereas extension IDP was significantly higher in the lower compared to the upper thoracic spine, both showing significant (*p* < 0.01) linear correlation with the segmental level at 7.5 Nm (flexion: *r* = −0.629, extension: *r* = 0.500). No significant effects of sex or age were detected, however trends toward higher IDP in specimens from female donors and decreasing IDP with increasing age, potentially caused by fibrotic degenerative changes in the nucleus pulposus tissue. Sagittal and transversal cuttings after testing revealed possible relationships between nucleus pulposus quality and pressure moment characteristics, overall leading to low or negative intrinsic IDP and non-linear pressure-moment behavior in case of fibrotic tissue alterations. In conclusion, this study provides insights into thoracic spinal IDP and offers a large dataset for the validation of numerical models of the thoracic spine.

## Introduction

The hydrostatic pressure of the nucleus pulposus plays a key role in the biomechanical properties of the thoracic spine, reducing compressive stress gradients in the anulus fibrosus (Stefanakis et al., 2014) and affecting the segmental stability more than any ligamentous or bony structure (Wilke et al., 2020). Several investigators explored the characteristics of intradiscal pressure in various *in vitro, in vivo*, as well as numerical studies, starting with Nachemson, who analyzed the intradiscal pressure in human lumbar spinal specimens (Nachemson, 1959, 1960). Since then, *in vitro* intradiscal pressure measurements were predominantly performed on the cervical (Pospiech et al., 1999; Dmitriev et al., 2005; Kretzer et al., 2012; Barrey et al., 2015; Liu et al., 2016; Welke et al., 2016; Bell et al., 2018) and lumbar spine (Wilke et al., 1996; Rohlmann et al., 2001; Molz et al., 2003; Schmoelz et al., 2006; Heuer et al., 2007; Gao et al., 2011). On the thoracic spine, in contrast, solely *in vivo* measurements in mid- and lower thoracic segmental levels (Polga et al., 2004) and few *in vitro* measurements on specific levels in polysegmental setups (Cheng et al., 2015; Anderson et al., 2016; Metzger et al., 2016) have been conducted so far. For the validation of numerical models and for ensuring high comparability of experimental setups, however, intradiscal pressure data from tests with clearly defined boundary conditions is essential. The purpose of this *in vitro* study therefore was to quantify the intradiscal pressure in human thoracic spine specimens under pure moment loading.

## Materials and Methods

### Specimens

**Figure 1:**
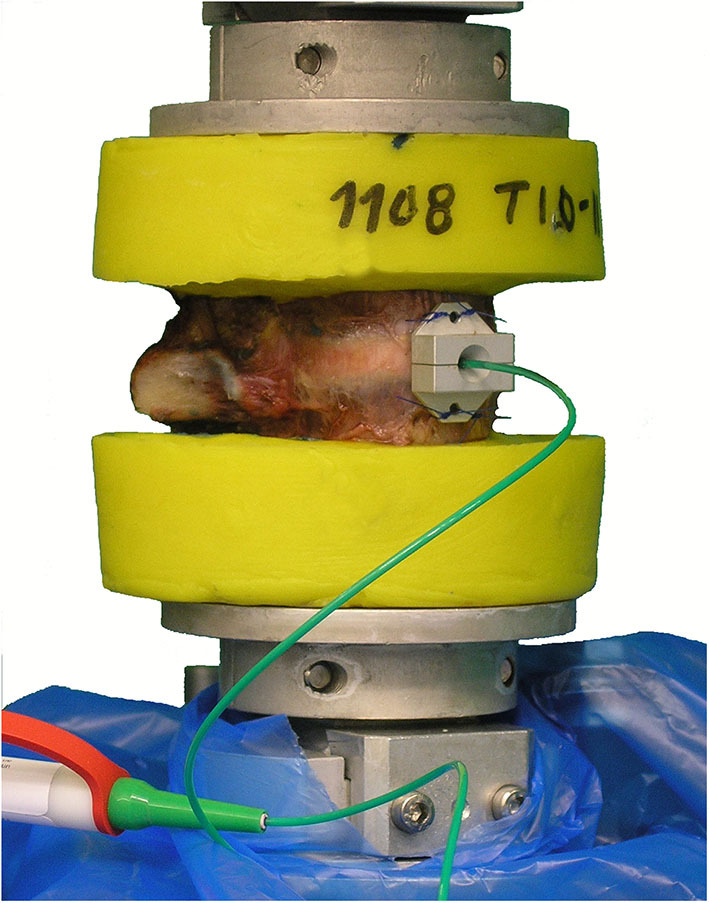
Illustration of the test setup, exemplarily showing a T10–T11 motion segment with implanted intradiscal pressure sensor prior to pure moment loading.

Thirty fresh-frozen human functional spinal units from 19 donors, including at least one specimen per thoracic spinal segmental level, were prepared for experimental testing. Nineteen specimens were from female donors and 11 from male donors, while average donor age was 56 years, ranging from 43 to 75 years (Table 1). Inclusion criteria were sufficient intervertebral disc height and the absence of severe osteophyte formation, bony, cartilaginous, or ligamentous defects, fractures and tumors, which was confirmed by frontal and sagittal x-rays (Faxitron 43805N, Hewlett Packard, Palo Alto, USA). Additionally, adequate flexibility of the motion segments was proved by manual control after preparation. Specimens were stored at −20°C and were thawed for 12–14 h prior to preparation and testing, which was kept below 20 h in total. During preparation, care was taken to preserve all biomechanically relevant structures including all bony, cartilaginous, and ligamentous structures as well as the costovertebral joints, while the ribs were shortened to stumps with a length of about 20–30 mm and all muscle, fat, and nerve tissue was removed. Both vertebrae of the functional spinal units were embedded half, coaxially, and parallel to the intervertebral disc in polymethylmethacrylate (PMMA, Technovit 3040, Heraeus Kulzer, Wehrheim, Germany), while the center of the vertebral body was tried to be positioned in the cylinder axis of the embedding. Using modeling clay prior to embedding, it was ensured to keep the intervertebral ligaments and facet joints movable. Additionally, screws were driven into the vertebral bodies in order to enhance rigid fixation in the embedding before molding. For biomechanical testing, flanges were fixed coaxially on the PMMA cylinders. Pressure sensors were inserted by injecting cannulae in the center of the anterior side of the intervertebral disc, while care was taken that the measurement port of the sensor was positioned in the center of the nucleus pulposus and pointed in cranial direction in all specimens. Correct positioning of the sensor was verified in all planes using a mobile x-ray system (Exposcop CB7-D, Ziehm, Nuremberg, Germany). For fixation of the pressure sensors, a clamping device was sutured to the anterior longitudinal ligament (Figure 1). In order to avoid specimen disintegration, tissue was kept moist using 0.9% saline solution during the process of preparation and testing.

**Table 1:** Data on donor age (categorized into ranges for anonymization) and segmental levels used for experimental testing.

**Donor no**.	**Age range in years**	**Segmental levels**
1	41–45	T1–T2, T6–T7
2	41–45	T6–T7, T10–T11
3	46–50	T1–T2
4	46–50	T2–T3
5	46–50	T11–T12
6	51–55	T10–T11
7	51–55	T7–T8
8	51–55	T3–T4, T8–T9, T10–T11
9	51–55	T4–T5, T8–T9
10	56–60	T2–T3, T6–T7
11	56–60	T11–T12
12	56–60	T3–T4, T7–T8
13	56–60	T6–T7
14	61–65	T3–T4
15	61–65	T9–T10, T11–T12
16	61–65	T10–T11
17	61–65	T7–T8, T9–T10
18	71–75	T5–T6, T9–T10, T11–T12
19	71–75	T9–T10

### Experimental Setup

Intradiscal pressure measurement was performed using a sensor device with a diameter of 1.2 mm (FMSPEZ50, MIPM GmbH, Hattenhofen, Germany), including a piezoelectrical micro-pressure sensor in its metal tip, exhibiting a maximum pressure of 5 MPa and an accuracy of 1 kPa. This method had already been successfully applied to the cervical (Pospiech et al., 1999) and lumbar spine (Wilke et al., 1996; Rohlmann et al., 2001; Schmoelz et al., 2006; Heuer et al., 2007) in previous *in vitro* studies. Before each testing, the sensors were calibrated up to 0.6 MPa using a (Digibar II, HBM GmbH, Darmstadt, Germany). Specimen loading was performed using a well-established spine tester, allowing six-degree-of-freedom quasi-static flexibility testing (Wilke et al., 1994). By means of this technique, range of motion and neutral zone values were already quantified for all thoracic spinal segmental levels in a previous *in vitro* study (Wilke et al., 2017).

### Testing Procedure

The specimens were loaded up to pure moments of 7.5 Nm with an angular velocity of 1°/s in flexion/extension and lateral bending as well as 0.5°/s in axial rotation. Each test run included 3.5 loading cycles per motion plane, while the third full loading cycle was extracted for data evaluation, while the first to loading cycles served for preconditioning of the intervertebral disc and the ligaments (Wilke et al., 1998). After biomechanical testing, the specimens were cut along the sagittal plane using a diamond band saw EXAKT Advanced Technologies GmbH, Norderstedt, Germany) in order to additionally assess the intervertebral disc quality.

### Data Evaluation and Statistics

**Figure 2:**
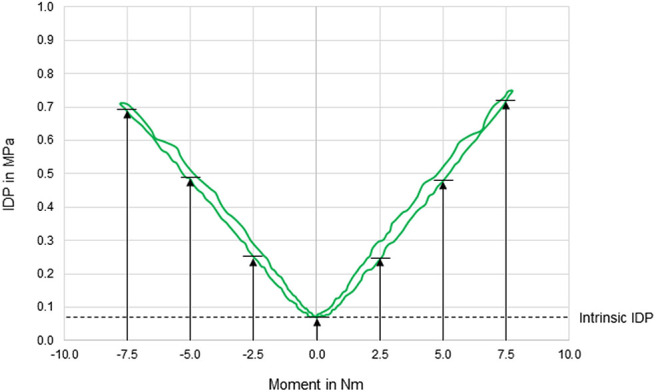
Exemplary diagram illustrating the intradiscal pressure (IDP) as a function of applied moment in lateral bending of a T11–T12 motion segment. IDP was quantified at pure moments of 0 Nm (intrinsic intradiscal pressure), 2.5, 5, and 7.5 Nm for each motion direction.

Intradiscal pressure and moment loading data was post-processed using Microsoft Excel 2016 (Microsoft Corp., Redmond, USA) and evaluated regarding pressure values at predefined pure moments of 0.0, 2.5, 5.0, and 7.5 Nm in all motion directions (Figure 2) using Matlab 2014b (MathWorks Inc., Natick, USA). Statistical significances and correlations were analyzed using SPSS 24 (IBM Corp., Armonk, USA). Two group comparisons (effect of sex) were performed using the Mann-Whitney *U*-test, while multiple group comparisons were conducted using either Friedman's ANOVA in case of dependent samples (differences between pressure values for same applied moments among all motion directions and between pressure values in every motion direction for all applied moments) or the Kruskal-Wallis test with Bonferroni-Dunn correction in case of independent samples (differences between thoracic spinal regions for same applied moments and motion directions), each with a significance level of 0.05. Linear correlation analysis was performed for the effect of segmental level and age on the intradiscal pressure using Pearson's r, while the significance level was set to 0.01. In case of significant correlation, linear regression analysis was additionally conducted to detect potential linear relationships between the respective parameters.

### Ethics, Funding, and Conflicts of Interest

The use of human specimens was approved by the ethical committee board of the University of Ulm, Germany, in November 2014 (No. 302/14). The specimens were acquired from the body donation organizations Anatomy Gifts Registry program (AGR, Hanover, Maryland, USA) and Science Care (Science Care Inc., Phoenix, Arizona, USA), which declared that written informed consent of the donors was obtained prior to decease. The study was funded by the German Research Foundation (DFG, WI1352/20-2). The authors declare to have no potential conflicts of interest.

## Results

**Figure 3:**
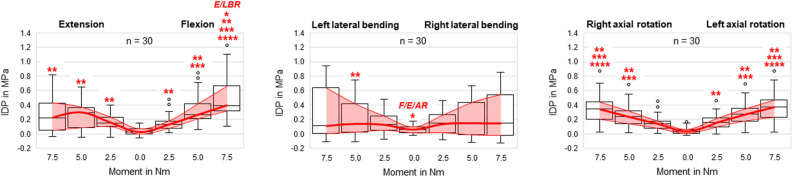
Boxplot diagrams illustrating the intradiscal pressure (IDP) values of all tested specimens (*n* = 30) as a function of the applied moment in the six motion directions, including medians, 25th and 75th percentiles, as well as maximum and minimum values. Outliers are plotted as points. The bold red line represents the connection between all median values, while the red area summarizes the medium 50-percentile zone. Significantly increased IDP values (*p* < 0.05) are marked by asterisks: ^*^, Significantly higher compared to respective motion directions with same applied moment (F, flexion; E, extension; LB(L/R), (left/right) lateral bending; AR(L/R), (left/right) axial rotation); ^**^, Significantly higher compared to intrinsic disc pressure at 0.0 Nm within the same motion direction; ^***^, Significantly higher compared to disc pressure at 2.5 Nm within the same motion direction; ^****^, Significantly higher compared to disc pressure at 5.0 Nm within the same motion direction.

In general, pressure-moment curves exhibited symmetrical, V-shaped characteristics (Figure 2), especially in axial rotation. Intradiscal pressure was generally highest in flexion movement at the pure moment of 7.5 Nm, which represented a significant increase compared to extension and right lateral bending for the same applied moment (Figure 3). Furthermore, the intradiscal pressure significantly increased for all applied moments compared to all previous steps in flexion direction, which was also found for bilateral axial rotation with the exception of the intradiscal pressure at 2.5 Nm in right axial rotation. Significant increases of the intradiscal pressure were also found for all loading steps in extension and at an applied moment of 5.0 Nm in left lateral bending, each compared to the intrinsic intradiscal pressure at 0.0 Nm. Median intrinsic pressure was below 0.06 MPa in all motion planes, while being significantly increased during lateral bending movement (0.058 MPa) compared to axial rotation (0.037 MPa) and flexion/extension (0.022 MPa). Variation range was generally lowest in axial rotation and flexion, while being highest in lateral bending, where the average v-shape of the pressure-moment curves was rather flattened. In extension, the median intradiscal pressure even tended to decrease from 5.0 to 7.5 Nm, leading to a non-linear pressure-moment behavior.

**Figure 4:**
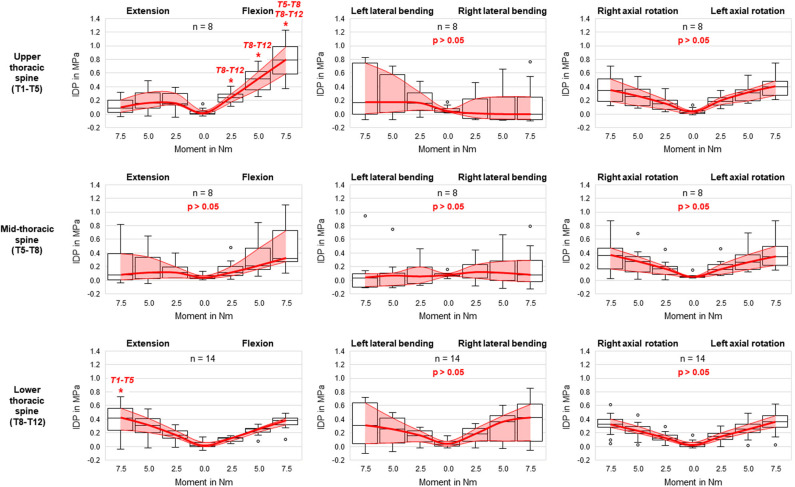
Boxplot diagrams illustrating the intradiscal pressure (IDP) values at upper, mid-, and lower thoracic spinal levels as a function of the applied moment in the six motion directions, including medians, 25th and 75th percentiles, as well as maximum and minimum values. Outliers are plotted as points. The bold red line represents the connection between all median values, while the red area summarizes the medium 50-percentile zone. Significantly increased IDP values (*p* < 0.05) compared to respective thoracic spinal regions within the same respective motion direction and with same applied moment are marked by ^*^.

**Figure 5:**
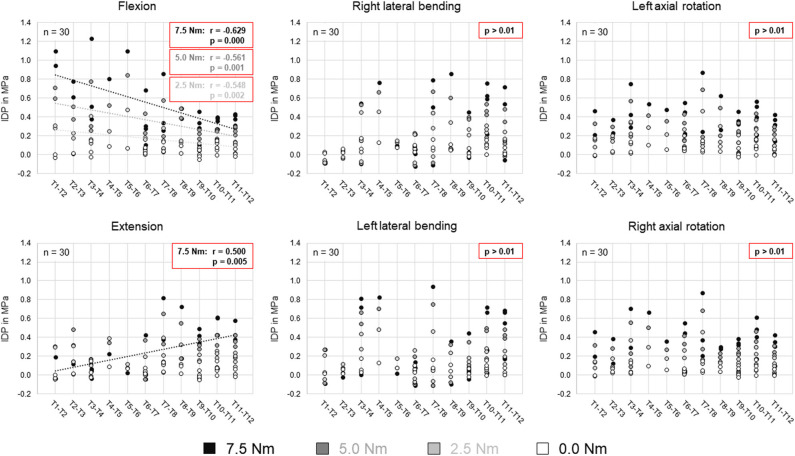
Scatter plots summarizing all intradiscal pressure (IDP) values as a function of the thoracic spinal segmental level for the four different applied moments in the six motion directions. Significant linear correlations (*p* < 0.01) are highlighted by dotted trend lines and the respective linear correlation coefficients.

Comparing the intradiscal pressure regarding thoracic spinal regions, significantly higher pressures were found in flexion of the upper thoracic spine (T1–T5) compared to the lower thoracic spine (T8–T12) for all loading steps as well as compared to the mid-thoracic spine (T5–T8) at the applied moment of 7.5 Nm (Figure 4). In contrast, the intradiscal pressure in extension was significantly higher in the lower thoracic spine compared to the upper thoracic spine at 7.5 Nm. Significant linear correlation was detected between thoracic spinal level in flexion for all applied moments and in extension at 7.5 Nm, while correlation was generally found being negative in flexion (*r* = −0.629/−0.561/−0.548 for 7.5/5.0/2.5 Nm, respectively) and positive in extension (*r* = 0.500) (Figure 5). Additional regression analysis exhibited an approximate intersection point of intradiscal pressure trend at T12 level with about 0.4 MPa in the flexion/extension motion plane. No significant differences between the single thoracic spinal regions or correlations between the single segmental levels and the intradiscal pressure were found in lateral bending and axial rotation. Variation range tended to be lowest in the lower thoracic spine in case of flexion and axial rotation movements, while it tended to be lowest in the mid-thoracic spine in case of lateral bending movements (Figure 4). In extension direction, the pressure-moment characteristics tended to alter in craniocaudal direction, with the median intradiscal pressure peak tending to shift toward higher applied moments.

**Figure 6:**
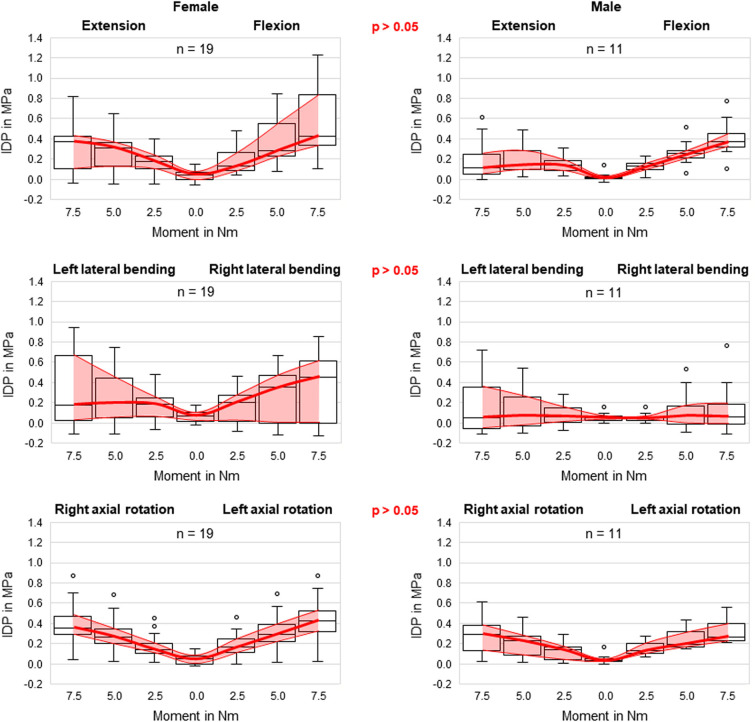
Boxplot diagrams illustrating the intradiscal pressure (IDP) values for specimens of female (left) and male (right) donors as a function of the applied moment in the six motion directions, including medians, 25th and 75th percentiles, as well as maximum and minimum values. Outliers are plotted as points. The bold red line represents the connection between all median values, while the red area summarizes the medium 50-percentile zone. Significant differences regarding sex were not detected in any motion direction.

**Figure 7:**
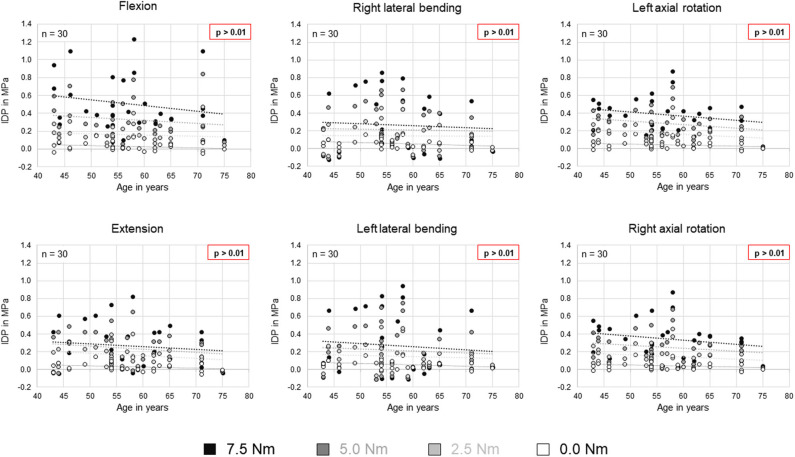
Scatter plots summarizing all intradiscal pressure (IDP) values as a function of donor age for the four different applied moments in the six motion directions. Significant linear correlations (*p* < 0.01) were not detected. Trends are illustrated by dotted lines.

**Figure 8:**
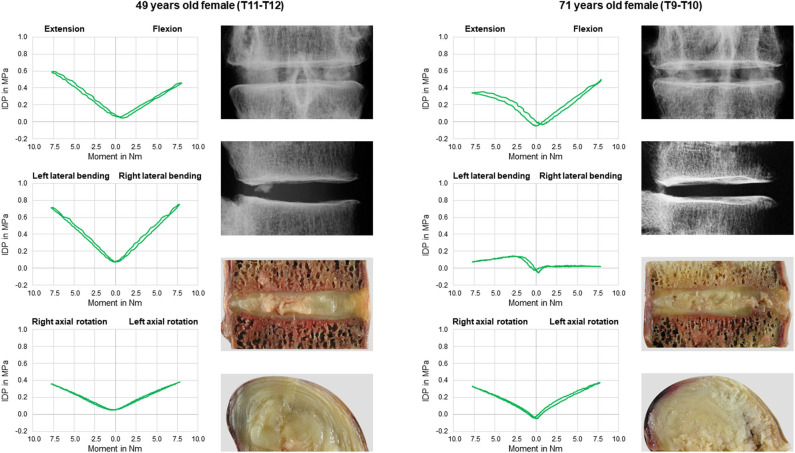
Exemplary comparison of pressure-moment characteristics in flexion/extension, lateral bending, and axial rotation (first and third row, top down) and intervertebral disc tissue quality in frontal and sagittal x-rays as well as sagittal and transversal cuttings (second and last row, top down) between a 49 years old female donor (left) and a 71 years old donor (right).

No statistical significant effects of sex and age on the intradiscal pressure were found in the present study. However, the intradiscal pressure tended to be higher and exhibited larger variation ranges in specimens from female donors compared to those of male donors (Figure 6). Moreover, slight trends were detected toward decreasing intradiscal pressure with increasing donor age for all applied moments in all motion directions (Figure 7). Although age had no statistically significant effect, indications were found that the intradiscal pressure was affected by the tissue quality of the intervertebral disc. While not showing substantial signs of degeneration in the x-ray images, fibrotic changes of the nucleus pulposus and the anulus fibrosus were especially detected in specimens from elderly donors, overall leading to low or even negative intrinsic pressure and more flattened pressure-moment characteristics compared to specimens from younger donors (Figure 8).

A summary of all evaluated parameters including the statistical analysis is given in the Supplementary Excel file attached to this publication.

## Discussion

The intradiscal pressure of the human thoracic spine has been poorly investigated. While several previous *in vivo* and *vitro* studies determined the intradiscal pressure of the cervical and lumbar spine, the hydrostatic pressure in thoracic intervertebral discs was solely explored in a single *in vivo* study (Polga et al., 2004). For a complete understanding of thoracic spinal biomechanics as well as for the accurate validation of *in vitro* and numerical models of the thoracic spine, however, data on the thoracic spinal intradiscal pressure is essential. The present *in vitro* study therefore aimed to quantify the intradiscal pressure of all human thoracic spine segmental levels under multi-planar pure moment loading.

Pressure-moment characteristics revealed a high dependence of the intradiscal pressure on the respective motion direction. While showing almost linear relationship in flexion and bilateral axial rotation, a distinct tendency was found in the upper and mid-thoracic spine toward a non-linear relationship in extension as well as in bilateral lateral bending, respectively. Since all segmental levels were tested under same loading conditions, the non-linear pressure-moment behavior in extension might indicate that in the upper and mid-thoracic spine, physiological loading is generally lower in extension direction compared to the other five motion directions when hypothesizing that the pressure-moment characteristics show linear behavior during elastic deformation of the intervertebral disc. However, due to the fact that the intradiscal pressure even decreased for higher moments in the upper segmental levels, it can be expected that the loads are rather transferred to the facet joints than to the intervertebral disc. Moreover, it was found that the flexion intradiscal pressure significantly decreased from the upper to the lower thoracic segmental levels, whereas the intradiscal pressure significantly increased, while being approximately equal at T12 level, suggesting that the intradiscal pressure divergence between flexion and extension generally decreases toward the thoracolumbar transition region. The pressure-moment characteristics in lateral bending, in contrast, exhibited a high variation range and low median intradiscal pressure values in the upper and mid-thoracic segmental levels, indicating a high impact of individual intervertebral disc properties on the lateral bending intradiscal pressure, such as degenerative, morphological, or biochemical features. In axial rotation, on the contrary, the pressure-moment characteristics were overall comparable across all segmental levels and revealed a low variation range, indicating a low effect of intervertebral disc properties on the intradiscal pressure during axial torsion. In general, thoracic spinal motion segments showed V-shaped pressure-moment behavior during entire loading cycles in all motion planes in the present study, while previous studies on the lumbar spine exhibited more butterfly-shaped pressure-moment characteristics (Wilke et al., 1996; Rohlmann et al., 2001; Schmoelz et al., 2006). This might indicate that in the thoracic spine, bending and torsion of the intervertebral disc leads to a more homogeneous volumetric compression of the nucleus pulposus compared to the lumbar spine, where the nucleus pulposus has a larger play due to higher deformability of the anulus fibrosus (Koeller et al., 1984). Therefore, the intervertebral disc height, which is greater in the lumbar spine compared to the thoracic spine (Pooni et al., 1986), might essentially contribute to the pressure-moment behavior.

In the present study, intrinsic intradiscal pressure values varied between −0.052 and 0.175 MPa with a median value of 0.037 MPa, while not exhibiting any significant effect of segmental level, sex, or age. However, significant effects of the motion plane were found, indicating potential effects of the motion plane on specimen preconditioning, although differences were overall minimal. Data of comparable *in vitro* studies on the lumbar spine also exhibited higher intrinsic intradiscal pressure in lateral bending compared to flexion/extension and axial rotation (Wilke et al., 1996; Rohlmann et al., 2001; Schmoelz et al., 2006), leading to the assumption that preconditioning in lateral bending might differ from the other two motion planes. Compared to previous *in vitro* studies on the cervical and lumbar spine, the intrinsic intradiscal pressure values of the present study showed a high variation range, while overall being in the same order of magnitude (Table 2). This large variation range might be explained by effects of intervertebral disc degeneration, which is known to increase with advancing age in the thoracic spine (Goh et al., 2000). Moreover, previous *in vitro* studies on the lumbar spine found that the intrinsic intradiscal pressure is lower in more degenerated intervertebral discs compared to healthy, young intervertebral discs, which generally show positive pressure values *in vitro* (Nachemson et al., 1979; Panjabi et al., 1988; Adams et al., 1996). As a consequence, it can be expected that disc degeneration induced the negative pressure values in the present study, although no distinct signs of degeneration were examined on the x-rays prior to the experiments. However, even mildly degenerated intervertebral discs exhibit hydrostatic properties, which was shown in a previous *in vitro* study on the lumbar spine (Adams et al., 1994). Therefore, specimens with negative pressure values were not excluded from the present study, since the primary aim was to evaluate the intradiscal pressure of average thoracic spinal motion segments. Nevertheless, the effect of intervertebral disc degeneration on the intradiscal pressure of the thoracic spine represents an important research question, which should be further investigated in future studies.

**Table 2:** Literature comparison between previous *in vitro* studies and the present *in vitro* study regarding intradiscal pressure (IDP) of human specimens without preload in different motion directions depending on segmental levels and applied moment.

**References**	**Level(s)**	**Intrinsic IDP (0 Nm) in MPa**	**Moment in Nm**	**Flexion IDP in MPa**	**Extension IDP in MPa**	**Lateral bending IDP in MPa**	**Axial rotation IDP in MPa**
Liu et al. (2016)^*^	C2–C6	–	1.5	0.2–0.3	0.1–0.2	0.2	0.2
Kretzer et al. (2012)^#^	C2–C3, C6–C7, C7–T1	–	2.0	0.1–0.4	0.1–0.4	0.1–0.5	–
Barrey et al. (2015)^*^	C3–C5	0.0–0.1	2.0	0.7	0.2–0.4	0.0–0.2	0.1–0.2
Pospiech et al. (1999)^#^	C3–C4, C5–C6	–	0.5	0.2–0.3	0.2–0.3	0.2	0.2–0.3
Bell et al. (2018)^*^	C4–C6	0.1–0.2	2.0	0.5–0.9	0.3–0.4	–	–
Welke et al. (2016)^*^	C4–C5, C6–C7	–	2.0	0.5	0.5	0.3	0.2
Dmitriev et al. (2005)^*^	C4–C5, C6–C7	–	5.0	0.5–1.1	0.5–1.1	0.3	0.2–0.3
Present study^#^	T1–T12	−0.1 to 0.2	2.5	0.1–0.5	0.0–0.4	−0.1 to 0.5	0.1–0.3
			5.0	0.2–0.8	0.0–0.4	−0.1 to 0.7	0.2–0.5
			7.5	0.3–1.1	0.0–0.5	−0.1 to 0.8	0.3–0.6
Metzger et al. (2016)	T7–T8	–	4.0	0.9	0.7	0.6	0.6
Gao et al. (2011)^*^	L3–L4, L5–S1	–	10.0	0.2	0.1	0.1	0.1
Schmoelz et al. (2006)^#^	L3–L4	0.1	10.0	0.3	0.3	0.3	0.2
Molz et al. (2003)^*^	L3–L4	–	10.0	0.1	0.1	0.0	0.1
Heuer et al. (2007)^#^	L4–L5	0.0	2.5	0.1	0.1	–	–
			5.0	0.2	0.1	–	–
			7.5	0.3	0.2	–	–
			10.0	0.3	0.2	–	–
Rohlmann et al. (2001)^*^	L4–L5	0.0–0.1	7.5	0.1	0.1	0.1	0.1
Wilke et al. (1996)^*^	L4–L5	0.1	3.75	0.2	0.2	0.2	0.2

*, Mean values reported;

#, *Median values reported*.

Previous investigations on the intradiscal pressure of the thoracic spine are limited to few *in vitro* studies using polysegmental test setups (Cheng et al., 2015; Anderson et al., 2016, 2018; Metzger et al., 2016) and one *in vivo* study (Polga et al., 2004). While Cheng et al. did not report absolute pressure values and Anderson et al. solely investigated the effect of follower loading on the intradiscal pressure at T4–T5 and T8–T9 levels, Metzger et al. found slightly higher average pressure values at T7–T8 level compared to the present study. Mean pressure values of Metzger et al., however, were within the variation range of the present study and exhibited similar pressure behavior with respect to the different motion directions, including pressure values being highest in flexion (Table 2). Anderson et al. (2018) furthermore identified increased pressure-moment slope after rib cage removal, indicating a stabilizing effect of the rib cage structures, which has to be considered when interpreting the results of the present study, where the anterior rib cage structures were neglected. Moreover, pressure-moment slopes were generally higher at T4–T5 level compared to T8–T9 level in all motion directions, which can be attributed to the use of an additional compressive follower load of 400 N in this study. In an *in vivo* study, Polga et al. measured the intradiscal pressure in mid- (T6–T8) and lower (T9–T11) thoracic spinal levels. They detected pressure values of about 0.3 MPa in the mid- and of about 0.2 MPa in the lower thoracic spine in prone position, presumably resulting from low muscular activity in axial compression direction, while in both upright sitting and standing, the intradiscal pressure increased to about 1.0 MPa in the mid- and to about 0.9 MPa in the lower thoracic spine. During bending and twisting movements of the participants, however, Polga et al. found increasing intradiscal pressure values from the mid- to the lower thoracic spine. Furthermore, the pressure values of Polga et al. were significantly higher compared to the values of a previous study on the lumbar spine in upright standing position, but significantly lower when compared to forward flexed position while holding a weight of 20 kg (Nachemson and Elfström, 1970). The same trend was also found compared to a further *in vivo* study on the lumbar spine with one participant (Wilke et al., 1999). Therefore, it might be concluded that *in vivo*, the intradiscal pressure decreases along the thoracolumbar spine in sole upright position, whereas the pressure increases along the thoracolumbar spine in bending or twisting positions. Comparing these findings with the combined data of the present *in vitro* study as well as previous *in vitro* studies, investigating intradiscal pressure in the cervical spine (Pospiech et al., 1999; Dmitriev et al., 2005; Kretzer et al., 2012; Barrey et al., 2015; Liu et al., 2016; Welke et al., 2016; Bell et al., 2018) and lumbar spine (Wilke et al., 1996; Rohlmann et al., 2001; Molz et al., 2003; Schmoelz et al., 2006; Heuer et al., 2007; Gao et al., 2011) without axial preload, a different intradiscal pressure distribution can be observed. While showing almost consistent intrinsic intradiscal pressure along the spine, the intradiscal pressure tends to decrease along the spine under pure moment application in all motion directions, especially when seen in relation to the applied moment (Table 2). This might indicate that additional axial preload alters the intradiscal pressure distribution along the spine, which should be considered in future experimental testing as well as in numerical model development. Moreover, additional compressive preload affects the pressure-moment characteristics, as previously shown for the lumbar spine (Rohlmann et al., 2001), especially leading to increased intrinsic pressure, which has to be taken into account when comparing the *in vitro* data of the present study to *in vivo* data and when validating numerical models. Testing without additional compressive load therefore represents a major limitation of the present study. Nevertheless, the data of the present study is essential for the validation of numerical models of the thoracic spine due to its clearly defined boundary conditions and therefore highly reproducible data. Previous investigations suggest a 400 N follower preload in order to create physiological intradiscal pressure values in the thoracic spine (Anderson et al., 2016), while producing potential, hardly controllable artifacts due to coupling and clamping effects at the same time. Moreover, follower load was shown to significantly decrease the flexibility and to alter the three-dimensional motion behavior of the thoracic spine (Liebsch et al., 2018), leading to additional boundary conditions for numerical models. Furthermore, preloading of the specimen might affect the intradiscal pressure measurement due to the distinct decrease of intervertebral disc height, which is lowest in the thoracic spine (Pooni et al., 1986). When using the herein presented data for the validation of a numerical model of the thoracic spine, the costovertebral joints have to be considered due to the presence of the posterior sections of the ribs in the present study. These were left intact because of their stabilizing effect on thoracic spinal motion segments (Liebsch et al., 2017) and thus their potential influence on the intradiscal pressure. Another limitation of the present study is that possible effects of the sensor entry point in the anterior part of the intervertebral disc cannot be fully excluded and may have caused small measuring artifacts especially in flexion direction. However, all tested specimens in the present study showed adequate anterior disc height, suggesting only minimal effects of the sensor entry point.

In conclusion, a large dataset regarding intradiscal pressure values of the human thoracic spine was generated. This dataset can be used as comparative data for future *in vitro* studies as well as for the validation process of numerical models of the thoracic spine.

## Data Availability Statement

All datasets presented in this study are included in the article/Supplementary Material.

## Ethics Statement

The use of human specimens was approved by the ethical committee board of the University of Ulm, Germany, in November 2014 (No. 302/14). The specimens were acquired from the body donation organizations Anatomy Gifts Registry program (AGR, Hanover, Maryland, USA) and Science Care (Science Care Inc., Phoenix, Arizona, USA), which declared that written informed consent of the donors was obtained prior to decease.

## Author Contributions

H-JW: funding acquisition, discussion, and manuscript review. AH: study design, specimen preparation, and experimental testing. KW: specimen preparation and experimental testing. CL: data analysis, discussion, and manuscript preparation. All authors contributed to the article and approved the submitted version.

## Conflict of Interest

The authors declare that the research was conducted in the absence of any commercial or financial relationships that could be construed as a potential conflict of interest.

## References

[B1] Adams M.A. Green T.P. Dolan P. (1994). The strength in anterior bending of lumbar intervertebral discs. Spine 19, 2197–2203. 10.1097/00007632-199410000-00014 7809754

[B2] Adams M. A. McNally D. S. Dolan P. (1996). 'Stress' distributions inside intervertebral discs. The effects of age and degeneration. J. Bone Joint Surg. Br. 78, 965–972. 10.1302/0301-620X.78B6.0780965 8951017

[B3] Anderson D. E. Mannen E. M. Sis H. L. Wong B. M. Cadel E. S. Friis E. A.. (2016). Effects of follower load and rib cage on intervertebral disc pressure and sagittal plane curvature in static tests of cadaveric thoracic spines. J. Biomech. 49, 1078–1084. 10.1016/j.jbiomech.2016.02.038 26944690 PMC4851616

[B4] Anderson D. E. Mannen E. M. Tromp R. Wong B. M. Sis H. L. Cadel E. S.. (2018). The rib cage reduces intervertebral disc pressures in cadaveric thoracic spines by sharing loading under applied dynamic moments. J. Biomech. 70, 262–266. 10.1016/j.jbiomech.2017.10.005 29106896 PMC6193557

[B5] Barrey C. Rousseau M. A. Persohn S. Campana S. Perrin G. Skalli W. (2015). Relevance of using a compressive preload in the cervical spine: an experimental and numerical simulating investigation. Eur. J. Orthop. Surg. Traumatol. 25, 155–165. 10.1007/s00590-015-1625-2 25845316

[B6] Bell K. M. Yan Y. Hartman R. A. Lee J. Y. (2018). Influence of follower load application on moment-rotation parameters and intradiscal pressure in the cervical spine. J. Biomech. 76, 167–172. 10.1016/j.jbiomech.2018.05.031 29929892

[B7] Cheng I. Sundberg E. B. Iezza A. Lindsey D. P. Riew K. D. (2015). Biomechanical determination of distal level for fusions across the cervicothoracic junction. Global Spine J. 5, 282–286. 10.1055/s-0035-1546418 26225276 PMC4516757

[B8] Dmitriev A. E. Cunningham B. W. Hu N. Sell G. Vigna F. McAfee P. C. (2005). Adjacent level intradiscal pressure and segmental kinematics following a cervical total disc arthroplasty: an *in vitro* human cadaveric model. Spine 30, 1165–1172. 10.1097/01.brs.0000162441.23824.95 15897831

[B9] Gao S. G. Lei G. H. He H. B. Liu H. Xiao W. F. Wen T.. (2011). Biomechanical comparison of lumbar total disc arthroplasty, discectomy, and fusion: effect on adjacent-level disc pressure and facet joint force. J. Neurosurg. Spine 15, 507–514. 10.3171/2011.6.SPINE11250 21780862

[B10] Goh S. Tan C. Price R. I. Edmondston S. J. Song S. Davis S.. (2000). Influence of age and gender on thoracic vertebral body shape and disc degeneration: an MR investigation of 169 cases. J. Anat. 197, 647–657. 10.1046/j.1469-7580.2000.19740647.x 11197538 PMC1468180

[B11] Heuer F. Schmidt H. Claes L. Wilke H. J. (2007). Stepwise reduction of functional spinal structures increase vertebral translation and intradiscal pressure. J. Biomech. 40, 795–803. 10.1016/j.jbiomech.2006.03.016 16712856

[B12] Koeller W. Meier W. Hartmann F. (1984). Biomechanical properties of human intervertebral discs subjected to axial dynamic compression. A comparison of lumbar and thoracic discs. Spine 9, 725–733. 10.1097/00007632-198410000-00013 6505843

[B13] Kretzer R. M. Hsu W. Hu N. Umekoji H. Jallo G. I. McAfee P. C. (2012). Adjacent-level range of motion and intradiscal pressure after posterior cervical decompression and fixation: an *in vitro* human cadaveric model. Spine 37, E778–E785. 10.1097/BRS.0b013e31824780b8 22228326

[B14] Liebsch C. Graf N. Appelt K. Wilke H. J. (2017). The rib cage stabilizes the human thoracic spine: An *in vitro* study using stepwise reduction of rib cage structures. PLoS ONE 12:e0178733. 10.1371/journal.pone.0178733 28570671 PMC5453693

[B15] Liebsch C. Graf N. Wilke H. J. (2018). The effect of follower load on the intersegmental coupled motion characteristics of the human thoracic spine: an *in vitro* study using entire rib cage specimens. J. Biomech. 78, 36–44. 10.1016/j.jbiomech.2018.06.025 30031651

[B16] Liu Q. Guo Q. Yang J. Zhang P. Xu T. Cheng X.. (2016). Subaxial cervical intradiscal pressure and segmental kinematics following atlantoaxial fixation in different angles. World Neurosurg. 87, 521–528. 10.1016/j.wneu.2015.09.025 26409072

[B17] Metzger M. F. Robinson S. T. Svet M. T. Liu J. C. Acosta F. L. (2016). Biomechanical analysis of the proximal adjacent segment after multilevel instrumentation of the thoracic spine: do hooks ease the transition? Global Spine J. 6, 335–343. 10.1055/s-0035-1563611 27190735 PMC4868576

[B18] Molz F. J. Partin J. I. Kirkpatrick J. S. (2003). The acute effects of posterior fusion instrumentation on kinematics and intradiscal pressure of the human lumbar spine. J. Spinal Disord Tech. 16, 171–179. 10.1097/00024720-200304000-00009 12679672

[B19] Nachemson A. (1959). Measurement of intradiscal pressure. Acta Orthop. Scand. 28, 269–289. 10.3109/17453675908988632 14425681

[B20] Nachemson A. (1960). Lumbar intradiscal pressure. Experimental studies on post-mortem material. Acta Orthop. Scand. Suppl. 43, 1–104. 10.3109/ort.1960.31.suppl-43.01 14425680

[B21] Nachemson A. Elfström G. (1970). Intravital dynamic pressure measurements in lumbar discs. A study of common movements, maneuvers and exercises. Scand J. Rehabil. Med. Suppl. 1, 1–40. 4257209

[B22] Nachemson A. L. Schultz A. B. Berkson M. H. (1979). Mechanical properties of human lumbar spine motion segments. Influence of age, sex, disc level, and degeneration. Spine 4, 1–8. 10.1097/00007632-197901000-00001 432710

[B23] Panjabi M. Brown M. Lindahl S. Irstam L. Hermens M. (1988). Intrinsic disc pressure as a measure of integrity of the lumbar spine. Spine 13, 913–917. 10.1097/00007632-198808000-00008 3187715

[B24] Polga D. J. Beaubien B. P. Kallemeier P. M. Schellhas K. P. Lew W. D. Buttermann G. R.. (2004). Measurement of *in vivo* intradiscal pressure in healthy thoracic intervertebral discs. Spine 29, 1320–1324. 10.1097/01.BRS.0000127179.13271.78 15187632

[B25] Pooni J. S. Hukins D. W. Harris P. F. Hilton R. C. Davies K. E. (1986). Comparison of the structure of human intervertebral discs in the cervical, thoracic and lumbar regions of the spine. Surg. Radiol. Anat. 8, 175–182. 10.1007/BF02427846 3099408

[B26] Pospiech J. Stolke D. Wilke H. J. Claes L. E. (1999). Intradiscal pressure recordings in the cervical spine. Neurosurgery 44, 379–384. 10.1097/00006123-199902000-00078 9932892

[B27] Rohlmann A. Neller S. Claes L. Bergmann G. Wilke H. J. (2001). Influence of a follower load on intradiscal pressure and intersegmental rotation of the lumbar spine. Spine 26, E557–E561. 10.1097/00007632-200112150-00014 11740371

[B28] Schmoelz W. Huber J. F. Nydegger T. Claes L. Wilke H. J. (2006). Influence of a dynamic stabilisation system on load bearing of a bridged disc: an *in vitro* study of intradiscal pressure. Eur. Spine J. 15, 1276–1285. 10.1007/s00586-005-0032-5 16429291 PMC3233955

[B29] Stefanakis M. Luo J. Pollintine P. Dolan P. Adams M. A. (2014). ISSLS Prize winner: mechanical influences in progressive intervertebral disc degeneration. Spine 39, 1365–1372. 10.1097/BRS.0000000000000389 24831499

[B30] Welke B. Schwarze M. Hurschler C. Book T. Magdu S. Daentzer D. (2016). *In vitro* investigation of a new dynamic cervical implant: comparison to spinal fusion and total disc replacement. Eur. Spine J. 25, 2247–2254. 10.1007/s00586-015-4361-8 26684468

[B31] Wilke H.J. Grundler S. Ottardi C. Mathew C.E. Schlager B. Liebsch C. (2020). In vitro analysis of thoracic spinal motion segment flexibility during stepwise reduction of all functional structures. Eur. Spine J. 29, 179–185. 10.1007/s00586-019-06196-7 31664565

[B32] Wilke H. J. Claes L. Schmitt H. Wolf S. (1994). A universal spine tester for *in vitro* experiments with muscle force simulation. Eur. Spine J. 3, 91–97. 10.1007/BF02221446 7874556

[B33] Wilke H. J. Herkommer A. Werner K. Liebsch C. (2017). *In vitro* analysis of the segmental flexibility of the thoracic spine. PLoS ONE 12:e0177823 10.1371/journal.pone.0177823 28520819 PMC5433776

[B34] Wilke H. J. Neef P. Caimi M. Hoogland T. Claes L. E. (1999). New *in vivo* measurements of pressures in the intervertebral disc in daily life. Spine 24, 755–762. 10.1097/00007632-199904150-00005 10222525

[B35] Wilke H. J. Wenger K. Claes L. (1998). Testing criteria for spinal implants: recommendations for the standardization of *in vitro* stability testing of spinal implants. Eur. Spine J. 7, 148–154. 10.1007/s005860050045 9629939 PMC3611233

[B36] Wilke H. J. Wolf S. Claes L. E. Arand M. Wiesend A. (1996). Influence of varying muscle forces on lumbar intradiscal pressure: an *in vitro* study. J. Biomech. 29, 549–555. 10.1016/0021-9290(95)00037-2 8964785

